# MIND diet intervention for cognitive function in new‐onset mild stroke: a pilot randomized controlled trial

**DOI:** 10.1002/alz70861_108236

**Published:** 2025-12-23

**Authors:** Changzheng Yuan, Hui Chen, Qing Lin, Leqi Fei, Jie Shen, Mengjia Zhao, Tao Zhang, Lili Tang, Klodian Dhana, Xiaoran Liu, Lusha Tong

**Affiliations:** ^1^ Zhejiang University School of Medicine, Hangzhou, Zhejiang China; ^2^ Harvard T. H. Chan School of Public Health, Boston, MA USA; ^3^ Tianjin Medical University, Tianjin, Tianjin China; ^4^ Rush Institute for Healthy Aging, Chicago, IL USA

## Abstract

**Background:**

Post‐stroke cognitive impairment and dementia (PSCID) are common among stroke survivors, yet effective preventive strategies remain limited. The potential cognitive benefits of Mediterranean‐DASH Intervention for Neurodegenerative Delay (MIND) diet for stroke survivors have not been fully revealed.

**Method:**

The MIND Diet to Improve Cognitive Function in Mild Stroke Patients (MINDICOMS, NCT05921084) was a 26‐week, two‐arm pilot randomized controlled trial evaluating the effects of the MIND diet on cognitive function in patients with new‐onset mild stroke. We enrolled 60 patients aged 35–70 years with acute ischemic stroke and signs of cognitive impairment without dementia. Participants were randomized 1:1 to receive either standard care plus a MIND diet intervention or standard care alone. The intervention group additionally received Chinese‐adapted MIND diet education, a 7‐day in‐hospital whole meal provision, and a 26‐week food supply. MIND diet adherence was assessed using food frequency questionnaires at baseline and Week 26. The primary efficacy outcome was change in global cognition, measured by a neuropsychological battery at baseline, Week 13, and Week 26. Mixed‐effects models were used to estimate between‐group differences over time.

**Result:**

Of the 60 participants (mean age 57.9 years, 30% female), 53 and 54 completed assessments at Weeks 13 and 26, respectively. From baseline to Week 26, the MIND diet group showed a significant increase in MIND diet score versus a minor change in the control group (2.24 vs. ‐0.07; between‐group difference: 2.31, 95% CI: 1.32 to 3.31, *p* < 0.001). At Week 13, the MIND diet group improved more in global cognition than the control group (mean change from baseline: 0.615 vs. 0.367; difference: 0.249, 95% CI: 0.071 to 0.427, *p* = 0.007), which was widened at Week 26 (0.837 vs. 0.531; difference: 0.307, 95% CI: 0.128 to 0.485, *p* = 0.001). Findings from the extended 52‐week follow‐up will be presented at the conference.

**Conclusion:**

A 26‐week MIND diet intervention improved diet quality and cognitive function in mild stroke patients. These findings support the feasibility and potential benefit of dietary strategies for cognitive health in stroke survivors and provide preliminary evidence supporting the need for future large‐scale trials.

